# Is abaloparatide more efficacious on increasing bone mineral density than teriparatide for women with postmenopausal osteoporosis? An updated meta-analysis

**DOI:** 10.1186/s13018-023-03595-x

**Published:** 2023-02-17

**Authors:** Pan Hong, Ruikang Liu, Saroj Rai, JiaJia Liu, YeMing Zhou, Yu Zheng, Jin Li

**Affiliations:** 1 Department of Orthopaedic Surgery, Union Hospital, Tongji Medical College, Huazhong University of Science and Technology, Wuhan, 430030 China; 2 Department of Endocrinology, Union Hospital, Tongji Medical College, Huazhong University of Science and Technology, Wuhan, China; 3 Department of Orthopaedics and Trauma Surgery, Dubai Investment Park Br, Karama Medical Center, Dubai, United Arab Emirates; 4 First Clinical School, Tongji Medical College, Huazhong University of Science and Technology, Wuhan, China; 5 Basic Medical School, Tongji Medical College, Huazhong University of Science and Technology, Wuhan, 430030 China

**Keywords:** Abaloparatide, Teriparatide, Osteoporosis, Postmenopausal women, Meta-analysis

## Abstract

**Purpose:**

Osteoporosis poses a challenge to public health, causing fragility fractures, especially in postmenopausal women. Abaloparatide (ABL) is an effective anabolic agent to improve bone formation and resorption among postmenopausal women with osteoporosis. Our meta-analysis aims to assess the effectiveness and safety of ABL versus teriparatide (TPTD) in improving bone mineral density (BMD).

**Methods:**

We searched Medline, Embase, Web of Science, Cochrane databases and Clinicaltrial.gov until September 2, 2022. We included data from randomized controlled trials (RCTs) and post hoc analyses of RCTs. Outcomes included BMD change from baseline and risks of adverse events. The Grading of Recommendations, Assessment, Development and Evaluation (GRADE) tool was used to evaluate the quality of outcomes.

**Results:**

Four studies including 16 subgroups were included in this study. In particular, RCTs with head-to-head comparisons of ABL and TPTD were used in the meta-analysis, and all were from manufacturer-sponsored trials. All parameters in 24 weeks except lumbar spine (versus TPTD) showed significant advantages in the ABL group. Only the results of two subgroups in ABL versus TPTD demonstrated *High* GRADE quality (femoral neck: weighted mean difference (WMD) = 1.58 [0.52, 2.63]; Total hip: WMD = 1.46 [0.59, 2.32]). However, our fracture data were insufficient. Besides, we found no evident difference in serious adverse events or deaths in either group and the incidence of hypercalcemia in the ABL group lessened by 51% compared with the TPTD group. Nevertheless, compared with placebo, ABL demonstrated higher risks of nausea and palpitations.

**Conclusion:**

ABL demonstrated a beneficial effect on BMD compared to both placebo and TPTD for postmenopausal women with osteoporosis. ABL also had insignificantly lowered adverse event risk than TPTD. ABL is an alternative for patients with postmenopausal osteoporosis.

**Supplementary Information:**

The online version contains supplementary material available at 10.1186/s13018-023-03595-x.

## Introduction

Osteoporosis poses a challenge to public health, causing causes fragility fractures, especially in postmenopausal women [1]. Fractures in the geriatric population, particularly hip fractures, are associated with a high risk of morbidity and mortality [2, 3]. Wright et al. [4] demonstrated that the prevalence of osteoporosis and low bone mass was 10.3% and 43.9%, respectively, in the US population over 50 years . The public spending for fragility fractures increases with lifestyle changes, population aging, urbanization, obesity, and birth cohort effects, which brings a tremendous economic burden on society [5, 6]. Besides, Si et al. [5] predicted that medical costs for major nodular bone fractures (wrist, vertebra and hip) in China would reach $19.92 billion and $25.43 billion by 2035 and 2050, respectively.

Osteoporosis treatment aims to improve bone quality and reduce fracture risks and adverse events [7, 8]. Lifestyle changes and nutritional supplements may be beneficial. Combining dietary protein, vitamin D and calcium supplementation with exercise has been reported as a safe and effective way to alleviate bone loss in postmenopausal women [9–11]. Pharmacological interventions mainly include antiresorptive therapy and anabolic therapy [7, 12]. Bisphosphonates, a typical antiresorptive medication, reduce the risks of hip fracture and osteoporotic vertebral compression fractures in most osteoporotic patients [13, 14]. However, its long-term application has been associated with osteonecrosis of the jaw and atypical femoral fracture [15]. On the other hand, anabolic agents are reserved for patients at very high risk of fractures, especially vertebral fractures [2]. In the past two decades, anabolic agents, including teriparatide (TPTD), abaloparatide (ABL) and romosozumab, have been approved by FDA, which represent a novel approach for treating osteoporosis by accelerating bone formation [16].

ABL, a synthetic parathyroid hormone–related peptide (PTHrP) analog with amino acid substitutions between positions 22 and 31 of PTHrP(1–34), is an alternative anabolic therapy for postmenopausal women with osteoporosis [17, 18]. Le et al. [19] derived from a discrete-event simulation model that ABL yielded higher quality-adjusted life years (QALYs) at a lower cost compared to TPTD treatment, with an incremental cost-effectiveness ratio of $333,266/QALY relative to placebo. The pros and cons of ABL have been reported, and it is still undergoing multiple clinical trials [17, 20]. Therefore, TPTD is the primary medication available in clinical settings, especially for patients at high risk of fractures [1–3]. This meta-analysis aims to evaluate the efficacy and safety of ABL and compare it with TPTD regarding its effects in women with postmenopausal osteoporosis.

## Methods

### Search strategy

Our review followed the guidelines of Preferred Reporting Items for Systematic Reviews and Meta-Analyses (PRISMA) (see Additional file 1: Appendix 1), and the protocol was registered in PROSPERO before the literature search. Two independent reviewers (YMZ and RL) searched Medline, Embase, Web of Science and Cochrane databases updated to July 10, 2021, for randomized controlled trials (RCTs) (we processed another search at the end of the study on Sep 2, 2022). To expand the search range, the keywords were "osteoporosis", "Abaloparatide" or "BA058" or “BIM-44058” or “ITM-058”, and "randomized controlled trial" or "clinical trial". Clinicaltrials.gov was searched for completed but unpublished results of RCTs. We used truncated terms for all fields and categorized study types as clinical trials or randomized controlled trials. The search strategy used for the Medline database is available as Additional material (see Additional file 1: Appendix 2). Two researchers (JJL and SR) independently screened the titles and abstracts, and articles meeting inclusion criteria were accessed for full-text review. They independently reviewed full-text articles for eligibility afterward, without language restriction. Reference lists of eligible reviews and trials were searched for additional citations. Any disagreement which could not be resolved by consensus would come to a third researcher PH for judgment.

### Selection criteria

Postmenopausal women patients with osteoporosis were included in our study, with no age, nationality and race restrictions. There was no restriction on dosage and administration method of ABL. Both intravenous and transdermal injections were included in the intervention group, and placebo treatment with identical appearance and TPTD treatment was regarded as the comparison group. We only analyzed data from RCTs rather than systematic reviews and retrospective studies. Other updated studies like post hoc analyses, which were directed to detailed subgroup data of some specific phase three RCTs with complex and substantial outcomes, were included in the discussion. Women with osteosarcoma or other bone diseases, radiation therapy, malabsorption, renal calculi, urinary calculi, and renal dysfunction were excluded. Included studies must be in accordance with the Declaration of Helsinki and approved by respective ethics committees. Written informed consents of patients were required.

### Data extraction

Two researchers (JJL and YMZ) independently extracted data from eligible articles. The extracted data included characteristics of study (author, year of publication, journal, publication type, objective, type of disease, inclusion criteria, exclusion criteria, administration method, exposure, follow-up and funding source), characteristic of the patient (number of participants and age), baseline BMD and outcome data (BMD change and adverse events). Decisions were made by consulting a third reviewer PH in the case of disagreements and failed consensus. When data was incomplete, the corresponding author would be contacted by email and invited to send additional information.

Outcomes were classified as primary outcomes and secondary outcomes. Primary outcomes included BMD change from baseline. Secondary outcomes included the proportion of adverse events (main types were defined as a prevalence ≥ 5% from RCT by Miller, including palpitations, nausea, hypercalciuria, headache and back pain) [21]. Fracture risk was only included in the discussion due to insufficient data.

### Quality assessment

Cochrane Risk of Bias Assessment Tool (CROBAT) was used by two researchers (RL and YZ) to assess the quality of included studies independently. CROBAT included "Random sequence generation", "Allocation concealment", "Blinding of participants and personnel", "Blinding of outcome assessment", "Incomplete outcome data", "Selective reporting" and "Other bias" (see Additional file 1: Appendix 3). Each question had 3 answers: "Low risk", "Moderate" and "High risk". According to the published information, researchers would assess the risk level of RCTs. The decision was reached by consulting a third reviewer, PH, in the case of disagreements and failed consensus. Publication bias was evaluated by funnel plots and further confirmed by Egger's test, and *P* ≤ 0.05 was considered the statistically significant risk of bias. We used the Grading of Recommendations, Assessment, Development and Evaluation (GRADE) tool to evaluate the quality of evidence for each outcome, which is widely used to assess the quality of outcomes in meta-analyses. The GRADE tool classified evidence of outcomes into "High", "Moderate", "Low" and "Very low". Each assessment could reduce or promote the level of quality. Specific rules were explained in Additional file 1: Appendix 3.

### Statistical analysis

Continuous data using the same scale would be summarized by weighted mean difference (WMD) with 95% confidence intervals (CIs), while using different scales would be measured by standard mean difference (SMD) with 95% CIs. Dichotomous data would be calculated by odds ratio (OR) with 95% CIs. All statistical tests were two-tailed, and *P* ≤ 0.05 was regarded as a significant difference. Heterogeneity in the result of the meta-analysis was assessed by means of Cochrane Q and I2 statistics with appropriate analysis models: I^2^ > 50% indicated high heterogeneity and a random-effects model would be used in these outcomes; I^2^ ≤ 50% was considered an acceptable heterogeneity and a fixed-effects model would be used instead. GRADE is based on Risk of Bias, Inconsistency, Indirectness, etc. and is rated High when the low risk criteria are met. See Additional file 1: Appendix 3 for detailed criteria.

Subgroup analysis would be carried out when detailed data was available. We only did subgroup analyses for the comparison group (placebo or TPTD), while subgroup analyses for the administration method and dose of ABL were not performed because of insufficient data. Sensitivity analysis was performed in the meta-analysis by excluding each study once at a time to check whether the effectiveness of the outcome was determined by individual studies. Small-study effects leading to potential reporting or publication bias were avoided by Egger's test. Review Manager 5.3 and STATA 16.0 were used in our study.

## Results

### Search results

**Fig. 1:**
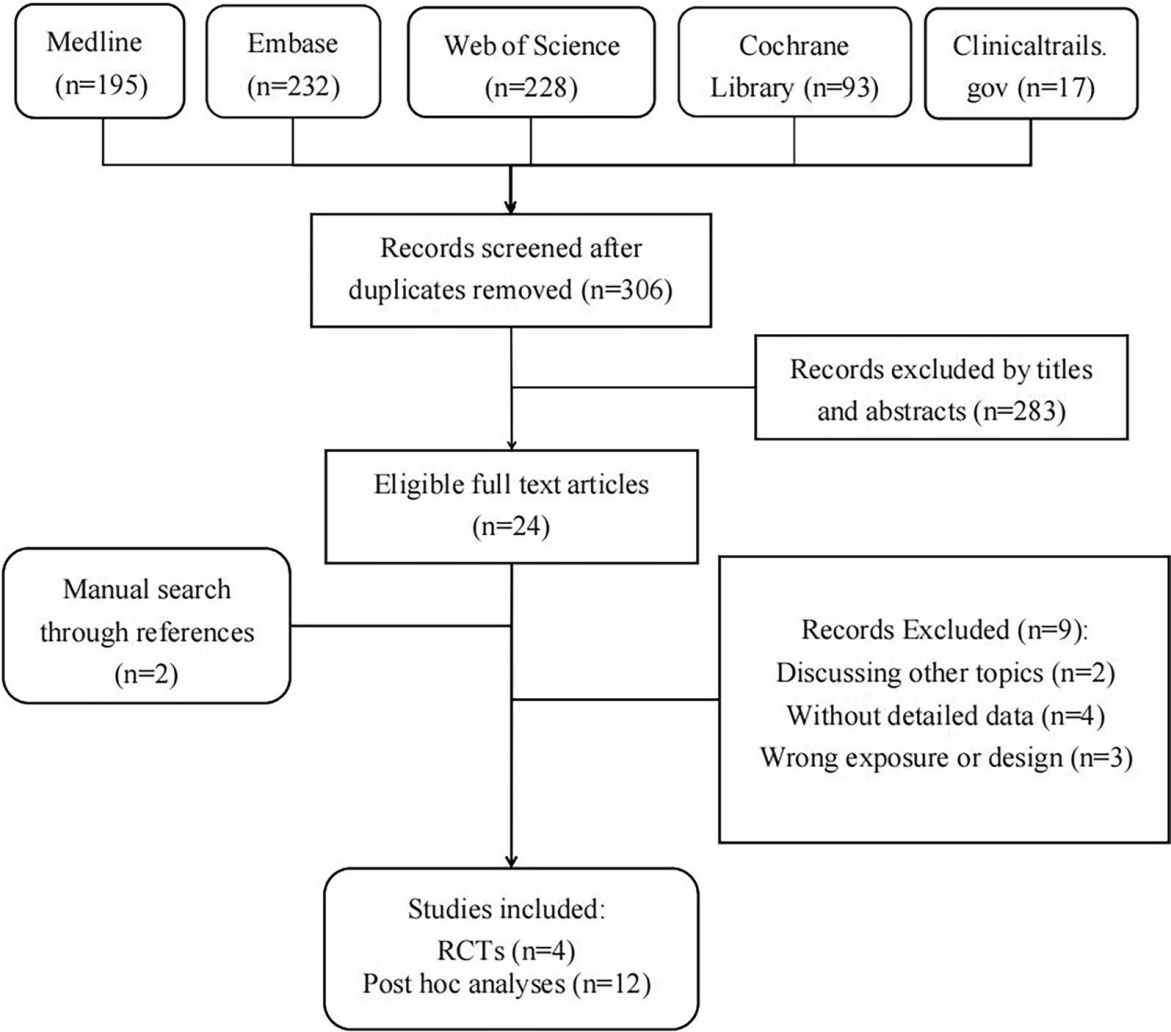
Flowchart of selection of included studies

Figure 1 demonstrates the detailed steps of the literature search. After retrieving 306 studies, 283 were screened out by browsing titles and abstracts, and the remaining 25 studies for full-text reviewing were conducted afterward. Subsequently, 9 studies were excluded by topic, data, study design or other selection criteria. Ultimately, 16 studies containing 2938 postmenopausal women, which included 4 RCTs and 12 post hoc analyses, were included in our meta-analysis (NCT01674621 had completed data on clinicaltrial.gov, but data were not published) [21–35]. The phase three RCT discussed by various post hoc analyses is Abaloparatide Comparator Trial In Vertebral Endpoints (ACTIVE) trial [21].

### Study characteristics

As shown in Table 1, all RCTs were multicenter trials: one of them was a phase-3 trial (*n* = 2463) and two of them were phase-2 trials (*n* = 222; *n* = 231). In terms of baseline BMD displayed in Additional file 1: Appendix 4, there was no significant difference in most comparisons, except for the result between ABL and TPTD in the femoral neck outcome (WMD = -− 0.01[− 0.02, 0.00], *P* = 0.04). Besides, only one study by Miller et al. contained data of the total hip [21]. We did not include a baseline BMD of the total hip for analysis in Additional file 1: Appendix 4.1 and 4.2 due to insufficient data. After discussion, we regarded this bias as negligible (< 5%). We also included 12 post hoc analyses [24–35], nine of which were conducted on the basis of clinical trial NCT01343004 [24–28, 31, 33–35], and the other three were conducted on the basis of clinical trial NCT01657162 [29, 30, 32]. These post hoc analyses focused on comparing ABL with TPTD or placebo alone and in combination and included studies of the amount of BMD change, fracture risk, and number needed to treat (NNT). Four RCTs and 12 post hoc analyses were all sponsored by Radius Health Inc. Moreover, we evaluated the risks of bias in our study that all RCTs are double-blinded and randomized (see Additional file 1: Appendix 5). Therefore, the qualities of included RCTs were high due to the strict design and complete results.

**Table 1:** Characteristics of studies included in systematic review

Author Year	Publication type	Exposure	Comparator	Number	Age	Follow-up	Funding
Leder 2015	Multicenter II-RCT (NCT00542425)	Abaloparatide: 20 µg (*n* = 43); 40 µg (*n* = 43); 80 µg (*n* = 45);	Placebo (*n* = 45); Teriparatide 20 μg (*n* = 45)	222	20 µg: 66.3 ± 7.0; 40 µg: 64.5 ± 7.4; 80 µg: 64.8 ± 7.2; Placebo: 65.0 ± 7.1; Teriparatide: 64.5 ± 7.5	48 weeks^1^	Radius Health Inc
Miller 2016	Multicenter III-RCT (NCT01343004)	Abaloparatide: 80 µg (*n* = 824)	Placebo (*n* = 821); Teriparatide 20 μg (*n* = 818)	2463	80 µg: 68.9 ± 6.5; Placebo: 68.7 ± 6.5; Teriparatide: 68.8 ± 6.6	72 + 4 weeks^2^	Radius Health Inc
Miller 2021*	Multicenter I-RCT (NCT04366726)	Abaloparatide*: 300 μg (*n* = 22)	/	22	300 μg: 65.2 ± 6.4	4 + 1 weeks^3^	Radius Health Inc
NCT01674621	Multicenter II-RCT (NCT01674621)	Abaloparatide#: 50 µg (*n* = 47); 80 µg (*n* = 49); 100 µg (*n* = 46); 150 µg (*n* = 43);	Placebo (*n* = 46)	231	50 µg: 65.9 ± 4.83; 80 µg: 66.4 ± 5.48; 100 µg: 65.7 ± 5.26; 150 µg: 66.3 ± 6.46; Placebo: 66.5 ± 7.27	24 + 4 weeks^4^	Radius Health Inc
Eastell 2019	Post hoc analysis^5^	Abaloparatide: 80 µg (*n* = 824)	Teriparatide 20 μg (*n* = 818)	1642	80 µg: 68.9 ± 6.5; Teriparatide: 68.8 ± 6.6	–	Radius Health Inc
McCloskey 2019	Post hoc analysis	Abaloparatide (*n* = 459)	Placebo (*n* = 468); Teriparatide (*n* = 473)	1400	Abaloparatide: 69.9 ± 6.67; Placebo: 70.0 ± 6.27; Teriparatide: 69.9 ± 6.37	–	Radius Health Inc
Reginster 2018	Post hoc analysis	Abaloparatide: 80 µg (*n* = 824)	Placebo (*n* = 821); Teriparatide 20 μg (*n* = 818)	2463	80 µg: 68.9 ± 6.5; Placebo: 68.7 ± 6.5; Teriparatide: 68.8 ± 6.6	–	Radius Health Inc
Leder 2019	Post hoc analysis	Abaloparatide 80 µg/Alendronate (*n* = 558)	Placebo/Alendronate (*n* = 581)	1139	80 µg: 68.9 ± 6.5; Placebo: 70.1 ± 6.3	–	Radius Health Inc
Reginster 2017	Post hoc analysis	Abaloparatide: 80 µg (*n* = 824)	Placebo (*n* = 821)	1645	80 µg: 68.9 ± 6.5; Placebo: 68.7 ± 6.5	–	Radius Health Inc
Bone 2018	Post hoc analysis	Abaloparatide 80 µg/Alendronate (*n* = 558)	Placebo/Alendronate (*n* = 581)	1139	80 µg: 68.9 ± 6.5; Placebo: 70.1 ± 6.3	–	Radius Health Inc
Cosman 2017	Post hoc analysis	Abaloparatide 80 µg/Alendronate (*n* = 558)	Placebo/Alendronate (*n* = 581)	1139	80 µg: 68.9 ± 6.5; Placebo: 70.1 ± 6.3	–	Radius Health Inc
Watts 2019	Post hoc analysis	Abaloparatide: 80 µg (*n* = 824)	Placebo (*n* = 821); Teriparatide 20 μg (*n* = 818)	2463	80 µg: 68.9 ± 6.5; Placebo: 68.7 ± 6.5; Teriparatide: 68.8 ± 6.6	–	Radius Health Inc
McCloskey 2017	Post hoc analysis	Abaloparatide: 80 µg (*n* = 824)	Placebo (*n* = 821)	1645	80 µg: 68.9 ± 6.5; Placebo: 68.7 ± 6.5	–	Radius Health Inc
Cosman 2016	Post hoc analysis	Abaloparatide: 80 µg (*n* = 824)	Placebo (*n* = 821)	1645	80 µg: 68.9 ± 6.5; Placebo: 68.7 ± 6.5	–	Radius Health Inc
Saag 2020	Post hoc analysis	Abaloparatide (*n* = 94)	Placebo (*n* = 103); Teriparatide (*n* = 99)	296	Abaloparatide: 59.4 ± 3.6; Placebo: 59.9 ± 3.3; Teriparatide: 58.8 ± 3.8	–	Radius Health Inc
McClung 2018	Post hoc analysis	Abaloparatide: 80 µg (*n* = 824)	Placebo (*n* = 821)	1645	80 µg: 68.9 ± 6.5; Placebo: 68.7 ± 6.5	–	Radius Health Inc

*Phase I study in which the drug was administered to the patient's thigh

^#^Abaloparatide Transdermal (50 µg/100 µg/150 µg); Abaloparatide Injection (80 µg)

^1^The patients in this RCT were treated for 48 weeks, but no follow-up time was given

^2^The patients in this RCT were treated for 72 weeks and had a follow-up of 4 weeks

^3^The patients in this RCT were treated for 4 weeks and had a follow-up of 1 weeks

^4^The patients in this RCT were treated for 24 weeks and had a follow-up of 4 weeks

^5^No follow-up period for post hoc analyses

### Primary outcome

**Fig. 2:**
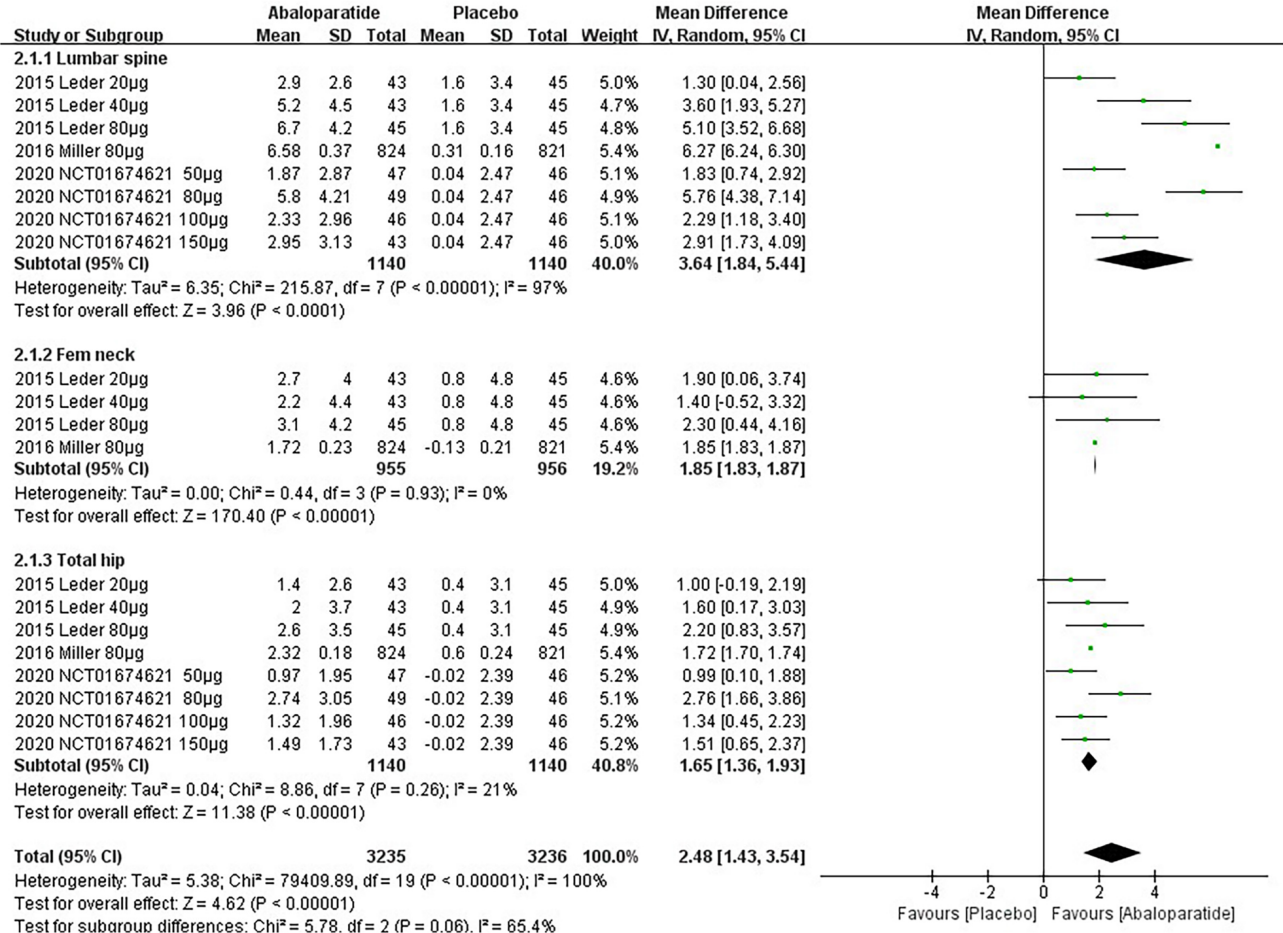
Forest plot of BMD Change (%)-24 weeks (Abaloparatide vs. Placebo)

**Fig. 3:**
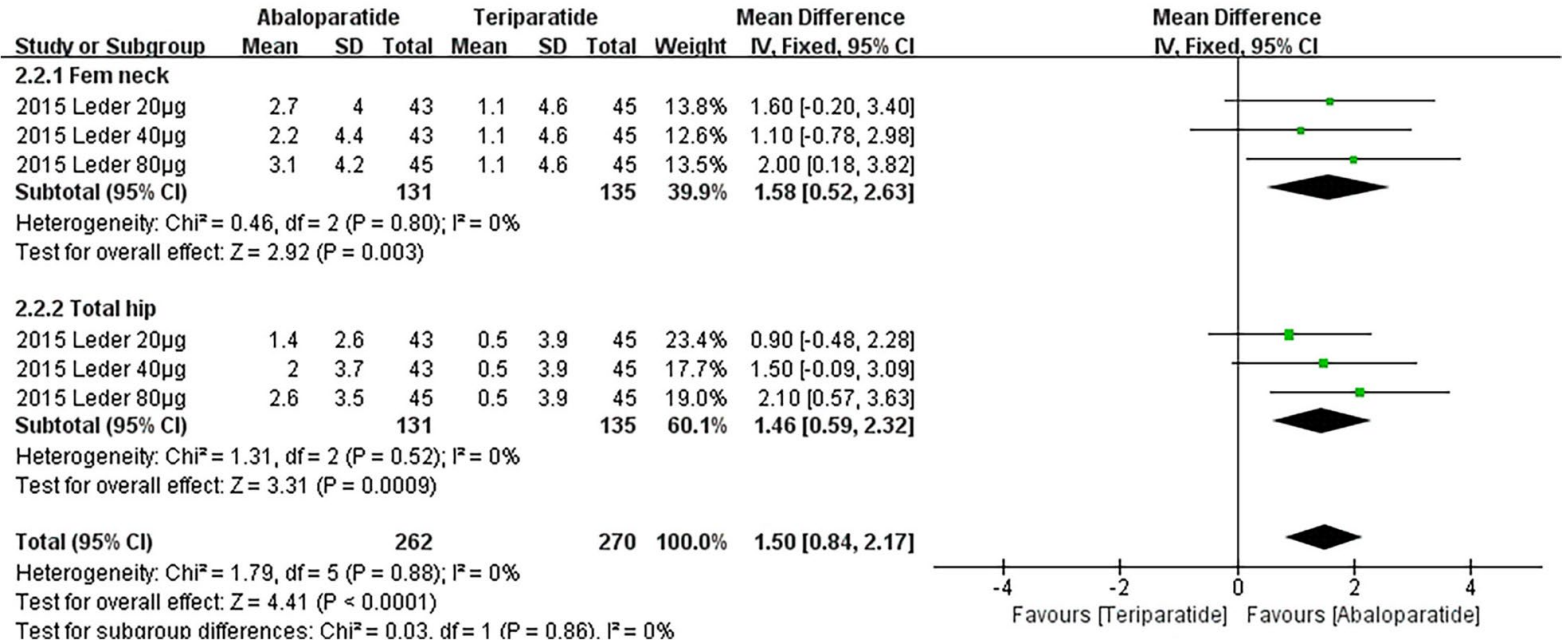
Forest plot of BMD Change (%)-24 weeks (Abaloparatide vs. Teriparatide)

Primary outcomes are listed in Table 2, which include the results of the time subgroup (24 weeks and 48 weeks) and BMD subgroups (lumbar spine, Fem neck and total hip). Due to the insufficient data in 48 weeks subgroup, we only show the detailed results of 24 weeks subgroup in Figs. 2 and 3. Figure 2 demonstrates all results of ABL versus placebo having a significant difference. Similarly, compared with TPTD, ABL also displays a significant advantage of BMD on femoral neck (WMD 1.58 [0.52, 2.63]) and total hip (WMD 1.46 [0.59, 2.32]) in Fig. 3. In the lumbar spine, ABL was less effective than TPTD (WMD − 0.61 [− 2.89, 1.68]). However, this result might be caused by low dose application, considering the high heterogeneity (I^2^ = 83%). Besides, on account of serious risks of heterogeneity and publication bias, only the results of two subgroups of ABL versus TPTD had a *High* grade.

**Table 2:** Primary outcome result of BMD change (%)**

BMD change (%)		No. of groups	Participants	Evidence synthesis	I^2^	*P* value	Egger's test	GRADE
*Abaloparatide versus Placebo*								
24 weeks	Lumbar spine	8	2280	WMD 3.64 [1.84, 5.44]	97%	*P* < 0.00001*	*P* < 0.0001^*^	Very Low
	Fem neck	4	1911	WMD 1.85 [1.83, 1.87]	0%	*P* < 0.00001*	*P* = 0.0003^*^	Low
	Total hip	8	2280	WMD 1.65 [1.36, 1.93]	21%	*P* < 0.00001*	*P* < 0.0001^*^	Low
48 weeks^#^	Lumbar spine	1	1645	WMD 9.32 [9.28, 9.36]	N/A	*P* < 0.00001*	N/A	Low
	Fem neck	1	1645	WMD 3.06 [3.04, 3.08]	N/A	*P* < 0.00001*	N/A	Low
	Total hip	1	1645	WMD 3.32 [3.30, 3.34]	N/A	*P* < 0.00001*	N/A	Low
*Abaloparatide versus Teriparatide*								
24 weeks	Lumbar spine	3	266	WMD -0.61 [− 2.89, 1.68]	83%	*P* = 0.60	*P* = 0.0007^*^	Very Low
	Fem neck	3	266	WMD 1.58 [0.52, 2.63]	0%	*P* = 0.003*	*P* = 0.8685	**High**
	Total hip	3	266	WMD 1.46 [0.59, 2.32]	0%	*P* = 0.0009*	*P* = 0.5417	**High**
48 weeks^#^	Lumbar spine	1	1642	WMD 1.49 [1.45, 1.53]	N/A	*P* < 0.00001*	N/A	Low
	Fem neck	1	1642	WMD 1.11 [1.08, 1.14]	N/A	*P* < 0.00001*	N/A	Low
	Total hip	1	1642	WMD 1.12 [1.10, 1.14]	N/A	*P* < 0.00001*	N/A	Low

Bold indicates a high level of confidence in the data

^#^Only "Miller 2016" (NCT01343004) has results for BMD change (%) at 48th week, which was administered as 80 μg of abaloparatide by daily injection

*These *P* values are less than or equal to 0.05

**The funnel plots are in the Additional file 1: Appendix 8

### Secondary outcome

Secondary outcomes are listed in Table 3. No evident difference was found in serious adverse events or deaths. In addition, the death rates of ABL were even less than placebo and TPTD. However, the comparison between ABL and placebo demonstrated significant differences in nausea and palpitations (Nausea: OR = 2.61 [1.73, 3.95]; Palpitations: OR = 12.54 [4.50, 34.93]). Besides, only one RCT reported nausea and palpitations, and similar conclusions appeared in the comparison between ABL and TPTD [23]. We also found the prevalence of hypercalcemia lessened by 51% in the ABL group versus TPTD, without significant difference (OR = 0.49 [0.18, 1.35]). Other adverse events showed no evident difference.

**Table 3:** Results of adverse events*

Adverse events	No. of groups	Participants	Evidence synthesis	I^2^	*P* value	Egger's test	GRADE
*Abaloparatide versus Placebo*							
Any adverse event	9	3443	OR 1.09 [0.91, 1.31]	0%	*P* = 0.33	0.6812	Moderate
Serious adverse events	9	3443	OR 0.91 [0.70, 1.19]	0%	*P* = 0.49	0.4012	Moderate
Deaths	2	2778	OR 0.47 [0.13, 1.68]	0%	*P* = 0.24	N/A	Moderate
Nausea	5	2044	OR 2.61 [1.73, 3.95]	0%	*P* < 0.00001	0.1555	**High**
Hypercalciuria	8	2314	OR 1.11 [0.83, 1.48]	0%	*P* = 0.47	0.0038	Very Low
Hypercalcemia	7	665	OR 1.30 [0.58, 2.91]	0%	*P* = 0.52	0.4974	Moderate
Headache	5	2044	OR 1.11 [0.79, 1.55]	0%	*P* = 0.55	0.0668	Moderate
Back pain	9	3443	OR 1.06 [0.81, 1.37]	0%	*P* = 0.69	0.0083	Very Low
Palpitations	5	2041	OR 12.54 [4.50, 34.93]	0%	*P* < 0.00001	0.1316	**High**
Abaloparatide versus Teriparatide							
Any adverse event	4	1908	OR 0.98 [0.75, 1.28]	0%	*P* = 0.87	0.0612	Moderate
Serious adverse events	3	1820	OR 0.99 [0.72, 1.37]	0%	*P* = 0.96	0.0125	Low
Deaths	2	2775	OR 0.64 [0.17, 2.46]	0%	*P* = 0.52	N/A	Low
Hypercalciuria	4	1908	OR 0.85 [0.64, 1.13]	0%	*P* = 0.27	0.1387	Moderate
Hypercalcemia	3	266	OR 0.49 [0.18, 1.35]	0%	*P* = 0.17	0.1056	**High**
Back pain	4	1908	OR 1.36 [0.94, 1.95]	5%	*P* = 0.10	0.2971	Moderate

Bold indicates a high level of confidence in the data

*The funnel plots are in the Additional file 1: Appendix 8

With limited information extracted from four RCTs, we only conducted subgroup analysis on ABL versus placebo and ABL versus TPTD. Other results with different subgroups from various data analyses were mentioned in the discussion section to compare with TPTD in more detail.

## Discussion

In our study, the ABL group demonstrated significant benefit of BMD change in all comparisons with placebo and better outcomes of BMD on femoral neck and total hip compared with the TPTD group in 24 weeks. As for adverse events, compared with the placebo, the ABL group demonstrated higher risks of nausea and palpitations, but the prevalence of hypercalcemia in the ABL group lessened by 51% versus TPTD.

Regarding fractures data, only Miller's study (NCT01343004) reported the percentage of fractures in three anatomic regions: new vertebral fractures, nonvertebral fractures and major osteoporotic fractures. Although we did not perform a uniform analysis, Miller’s study demonstrated that ABL reduced fracture rates in all areas compared to TPTD (new vertebral fracture: ABL: 4/824, TPTD: 6/818; nonvertebral fracture: ABL: 18/824, TPTD: 24/818; major osteoporotic fracture: ABL: 10/824, TPTD: 23/818).

Detailed types and recommended dosages were listed in Additional file 1: Appendix 6 [12, 20]. Alendronate, commonly applied in corticosteroid-induced osteoporosis treatment, is the cheapest and does not require subcutaneous administration. However, atypical femoral fractures (i.e., subtrochanteric fractures and focal lateral cortical thickening) and osteonecrosis of the jaw are two rare but severe side effects [15, 38]. Another antiresorptive drug, denosumab, is a fully human monoclonal antibody to the receptor activator of nuclear factor-kappa B ligand, which could decrease bone resorption and increase bone density. In a recent network meta-analysis, denosumab was shown to increase BMD in postmenopausal women with osteoporosis, with the best effect on the total hip and femoral neck [39]. Denosumab had also been reported as a beneficial treatment in preventing vertebral and hip fractures [40]. Still, cessation of denosumab is followed by rapid bone loss and an increase in the rate of vertebral fractures [41, 42]. We also include hormone replacement therapies in Additional file 1: Appendix 6, such as selective estrogen receptor modulators. Anabolic agents, TPTD (a recombinant form of PTHrP(1–34)) and ABL (a similar synthetic form of the PTHrP), binding to the PTH-1 receptor, have been compared by Bhattacharyya et al. (2019) on the mechanism at molecular level [43]. They pointed out that receptor activator of nuclear factor κB ligand (RANKL), a potent resorption inducer from osteoblastic cells, was caused by TPTD rather than PTHrP and increased RANKL production causes resorption. Besides, PTHrP had an osteogenic effect accompanied by lesser resorptive and hypercalcemic effects than TPTD because faster PTHrP-PTH1R dissociation and multiple substitutions between amino acids 22–34 of PTHrP were made to enhance the stability of peptides. Therefore, abaloparatide was more stable and overcame the loss of the anabolic window and hypercalcemia associated with TPTD. Moreover, romosozumab is a humanized monoclonal antibody to sclerostin resulting in an increase in bone formation and bone mineral density. Meanwhile, romosozumab has been reported to demonstrate consistent efficacy and similar safety profile in mild to moderate chronic kidney disease.[20, 44]. But Bilezikian et al. (2019) concluded that ABL demonstrated a beneficial result for patients whose estimated glomerular filtration rate was < 60 mL/min (3.6% for ABL versus 10.9% for TPTD) and had no evident negative effect on patients with renal impairment [45].

Detailed discussions below provided other subgroup analyses of ABL. Phase I RCT reported by Miller et al. in 2021 suggested ABL group demonstrated more effective outcomes than other two groups (ABL: 0.58% [*n* = 4]; placebo: 4.22% [*n* = 30]; TPTD: 0.84% [*n* = 6]) [23]. They also provided an alternative method of intradermal administration, the ABL-solid microstructured transdermal system, which resulted in successful efficacy and safety by self-administration. Besides data from ACTIVE, McClung et al. (2018) demonstrated that ABL improved BMD in elderly female patients (over 80 years old), and Saag et al. (2020) reported that ABL reduced fracture risks in younger menopausal female patients (49 to 64 years old) [24, 25]. Similarly, Cosman et al. (2017) and McCloske et al. (2017) proved that ABL provided protection against fractures consistently across various ages, BMD and fracture risks [26, 27]. Moreover, as for the subgroup of BMD measurement site, Watts et al. [28] suggested that ABL increased forearm BMD and decreased the risk of wrist fracture compared with the placebo or TPTD group.

The extended trial of ACTIVE (ACTIVExtend trial) concluded that 24 months of oral alendronate treatment after 18 months of subcutaneous ABL treatment was more effective than 24 months of alendronate treatment after 18 months of placebo in reducing the risk of fractures (87%, 52%, 45%, 58% reduction in vertebral, nonvertebral, clinical and major osteoporotic fractures compared to the placebo group respectively) [29]. The ACTIVExtend trial also reported substantial increases in ABL group on BMD at lumbar spine, femoral neck and total hip (ABL vs. Placebo: lumbar spine: 12.8% vs. 3.5%; total hip: 5.5% vs. 1.4%; femoral neck: 4.5% vs. 0.5%). In addition, patients during the ACTIVExtend trial displayed a similar situation of adverse events as ACTIVE [30, 31]. Moreover, initial treatment with ABL may result in greater vertebral fracture reduction compared with alendronate in postmenopausal women with osteoporosis [32]. In all, the ACTIVExtend trial suggested the therapeutic effect of ABL was long-lasting and safe.

The aforementioned studies verified the efficacy and safety of ABL for women with postmenopausal osteoporosis. Moreover, comparison between ABL and TPTD have been discussed in various studies with new assessment tools. Calculating the NNT to prevent one fracture is a new method to evaluate the efficacy of medicines. Based on the NNT data, ABL is a more effective treatment than TPTD both in vertebral (28 for ABL vs.30 for TPTD), nonvertebral (55 for ABL vs. 92 for TPTD), clinical (37 for ABL vs. 59 for TPTD) and major osteoporotic fractures (34 for ABL vs. 75 for TPTD) [33]. Moreover, the Committee for Medicinal Products for Human Use (CHMP) provided a risk threshold as guidance for new drugs for the treatment of primary osteoporosis [36]. Under the guidance of the CHMP, ABL showed the greater reduction of fracture risk than TPTD (morphometric vertebral fractures: 2 for ABL vs. 6 for TPTD; Nonvertebral fractures: 10 for ABL vs. 18 for TPTD; clinical fractures: 13 for ABL vs. 25 for TPTD) [34, 36]. In addition, bone alkaline phosphatase, *N*-terminal propeptide of type I procollagen, C-telopeptide of type I collagen and urinary cross-linked *N*-telopeptides of type I collagen are bone formation and resorption markers, which serve as bone turnover markers (BTM) [37, 46, 47]. Eastell et al. [35] compared the efficacy through BTM and found the increase of ABL in total hip and femoral neck BMD was greater than TPTD.

Cosman et al. concluded that TPTD, ABL and Romosozumab are effective agents for women with postmenopausal osteoporosis, but comparative investigation for optimal choice is warranted [48]. Our study provides insights from the perspective of BMD and complications. Several recent studies corroborated the results of our meta-analysis. In terms of fractures, McClung et al. (2018) demonstrated that ABL reduced the risk of vertebral and nonvertebral fractures [49]. Reginster et al. [50] also showed that ABL reduced the risk of wrist fractures in addition to vertebral and nonvertebral fractures. Our meta-analysis concluded that ABL demonstrated a beneficial effect on BMD change compared to TPTD and placebo. Yang et al. [51] also noted that ABL improved BMD in the lumbar spine and total hip. Besides, Hernandez et al. [52] concluded that ABL was better than TPTD in improving BMD but still not as good as romosozumab. In contrast, Tan et al. [53] summarized that there was no statistical difference between ABL, TPTD or romosozumab and placebo in safety. The reason for this difference may be that they looked at adverse events and serious adverse events, whereas our meta-analysis focused on the comparison of each different adverse event and presented more specific results.

FDA approved ABL as an alternative for postmenopausal women with osteoporosis in 2017 based only on data from Radius Health, Inc. [54]. We summarized important clinical indications and contraindications of ABL as follows: for adults, the daily subcutaneous injection dose is 80 µg; total duration should not exceed 24 months in a patient's lifetime; postmenopausal women with osteoporosis at high and multiple risks for fracture, or patients who have failed or are intolerant to other available anti-osteoporosis therapy are recommended for ABL treatment; ABL is not allowed in patients with primary or secondary hyperparathyroidism, hypercalcemia, palpitations, urinary calculi, osteosarcoma, and other (e.g., history of Paget's disease). Specific indications for the baseline of BMD and fracture history are shown in Additional file 1: Appendix 7.

A network meta-analysis in 2020 compared the efficacy of ABL with eight other drugs in reducing fracture risk. It suggested that ABL demonstrated better efficacy compared with other available drugs, such as TPTD, zoledronic acid and romosozumab [55]. However, Cosman et al. in YEAR reported inconsistent outcomes. Cosman et al. concluded that there was no significant difference in the effects of ABL, TPTD, and romosozumab. Therefore, the superiority of ABL remains controversial [48]. The strength of this study was its broad data source with comparisons between various treatments. However, its limitations were evident with only 2 RCTs of ABL with data on fracture risk (all RCTs were published before 2017), without subgroup analysis beyond the fracture site. Adverse events and costs were not discussed in this network meta-analysis as well. In contrast, our study is the first meta-analysis focusing solely on ABL for women with postmenopausal osteoporosis. We included more RCTs and data analyses with up-to-date data of ABL from high-quality RCTs, which reduced the potential bias. Besides, BMD was not discussed in the network meta-analysis but was regarded as our primary outcome with detailed analysis. Moreover, adverse events were only mentioned in their discussion without the specificity of an adverse event, while we selected seven types of adverse events for analysis and discovered certain significant differences. Furthermore, we introduced new assessment models and subgroup analyses for different administration, ages, baseline BMD and bone sites. In terms of data extraction, fracture risk is affected by multiple factors and is limited by short follow-up. Therefore, we regard BMD as our primary outcome, which is more objective and direct.

However, there were certain limitations in our study. Only four manufacturer-initiated RCTs (NCT00542425, NCT01343004, NCT04366726, and NCT01674621) with a total of 2938 patients were included in this study. The small number of RCTs inevitably limited the criteria of statistical analysis that we regarded different dosages as subgroups in the same forest plot of meta-analysis. Therefore, different ABL agents’ doses (20/40/50/80/100/150/300 µg) and administration duration (4/24/48/72 weeks) might lead to biases and impact its reliability. In addition, more RCTs of ABL are in progress without available data. According to clinicaltrial.gov, five RCTs of ABL are still recruiting (NCT03841058, NCT04167163, NCT04249232, NCT04467983, NCT03746041) and one RCT has been completed without publication (NCT04936984). Due to the limited available data, our study might be biased to a certain extent. In the future, academic centers without any conflicts of interest with the ABL manufacturer need to be selected for discussing optimal dosage and administration duration.

## Conclusion

ABL demonstrated a beneficial effect on BMD compared to both placebo and TPTD for postmenopausal women with osteoporosis. ABL also had insignificantly lowered adverse event risk than TPTD. We believe ABL is an alternative for postmenopausal women with osteoporosis.

## Supplementary Information

**Additional file 1:** **Appendix 1** PRISMA Checklist. **Appendix 2** Search Strategy of Medline. **Appendix 3** Assessment tools in Meta-analysis. **Appendix 4** Result of baseline BMD. **Appendix 5** Assessment result of CROBAT. **Appendix 6** Some Drugs for Postmenopausal Osteoporosis. **Appendix 7** Indications for the use of Abaloparatide. **Appendix 8** Funnel plots

## Abbreviations

PRISMA: Preferred reporting items for systematic reviews and meta-analyses
GRADE: Grading of recommendations, assessment, development and evaluation
QALYs: Quality-adjusted life years
RCTs: Randomized controlled trials
BMD: Bone mineral density
CROBAT: Cochrane risk of bias assessment tool
WMD: Weighted mean difference
CI: Confidence interval
SMD: Standard mean difference
OR: Odds ratio
PTHrP: Parathyroid hormone-related protein
RANKL: Receptor activator of nuclear factor κB ligand
ACTIVE: Abaloparatide comparator trial in vertebral endpoints
NNT: Number needed to treat
CHMP: Committee for medicinal products for human use
BTM: Bone turnover markers
