# Supplementary material for: Is abaloparatide more efficacious on increasing bone mineral density than teriparatide for women with postmenopausal osteoporosis? An updated meta-analysis
Source: J Orthop Surg Res. 2023 Feb 17;18:116. doi: 10.1186/s13018-023-03595-x (PMC9936648; doi:10.1186/s13018-023-03595-x)

**Supplemental files**

Appendix 1. PRISMA Checklist

Appendix 2. Search Strategy of Medline

Appendix 3. Assessment tools in Meta-analysis

Appendix 4. Result of baseline BMD

Appendix 5. Assessment result of CROBAT

Appendix 6. Some Drugs for Postmenopausal Osteoporosis

Appendix 7. Indications for the use of Abaloparatide

Appendix 8. Funnel plots of outcomes

Appendix 1. PRISMA Checklist

| **Section/Topic** | **#** | **Checklist Item** | **Reported on Page #** |
| --- | --- | --- | --- |
| **TITLE** | | | |
| Title | 1 | Identify the report as a systematic review, meta-analysis, or both. | 1 |
| **ABSTRACT** | | | |
| Structured summary | 2 | Provide a structured summary including, as applicable: background; objectives; data sources; study eligibility criteria, participants, and interventions; study appraisal and synthesis methods; results; limitations; conclusions and implications of key findings; systematic review registration number. | 2 |
| **INTRODUCTION** | | | |
| Rationale | 3 | Describe the rationale for the review in the context of what is already known. | 4 |
| Objectives | 4 | Provide an explicit statement of questions being addressed with reference to participants, interventions, comparisons, outcomes, and study design (PICOS). | 4 |
| **METHODS** | | | |
| Protocol and registration | 5 | Indicate if a review protocol exists, if and where it can be accessed (e.g., Web address), and, if available, provide registration information including registration number. | 5 |
| Eligibility criteria | 6 | Specify study characteristics (e.g., PICOS, length of follow-up) and report characteristics (e.g., years considered, language, publication status) used as criteria for eligibility, giving rationale. | 5 |
| Information sources | 7 | Describe all information sources (e.g., databases with dates of coverage, contact with study authors to identify additional studies) in the search and date last searched. | 5 |
| Search | 8 | Present full electronic search strategy for at least one database, including any limits used, such that it could be repeated. | 5 |
| Study selection | 9 | State the process for selecting studies (i.e., screening, eligibility, included in systematic review, and, if applicable, included in the meta-analysis). | 6 |
| Data collection process | 10 | Describe method of data extraction from reports (e.g., piloted forms, independently, in duplicate) and any processes for obtaining and confirming data from investigators. | 7 |
| Data items | 11 | List and define all variables for which data were sought (e.g., PICOS, funding sources) and any assumptions and simplifications made. | None |
| Risk of bias in individual studies | 12 | Describe methods used for assessing risk of bias of individual studies (including specification of whether this was done at the study or outcome level), and how this information is to be used in any data synthesis. | 7 |
| Summary measures | 13 | State the principal summary measures (e.g., risk ratio, difference in means). | 7 |
| Synthesis of results | 14 | Describe the methods of handling data and combining results of studies, if done, including measures of consistency (e.g., I^2^) for each meta-analysis. | 7 |
| Risk of bias across studies | 15 | Specify any assessment of risk of bias that may affect the cumulative evidence (e.g., publication bias, selective reporting within studies). | 7 |
| Additional analyses | 16 | Describe methods of additional analyses (e.g., sensitivity or subgroup analyses, meta-regression), if done, indicating which were pre-specified. | 8 |
| **RESULTS** | | | |
| Study selection | 17 | Give numbers of studies screened, assessed for eligibility, and included in the review, with reasons for exclusions at each stage, ideally with a flow diagram. | 8 |
| Study characteristics | 18 | For each study, present characteristics for which data were extracted (e.g., study size, PICOS, follow-up period) and provide the citations. | 8 |
| Risk of bias within studies | 19 | Present data on risk of bias of each study and, if available, any outcome level assessment (see item 12). | 8 |
| Results of individual studies | 20 | For all outcomes considered (benefits or harms), present, for each study: (a) simple summary data for each intervention group (b) effect estimates and confidence intervals, ideally with a forest plot. | 9 |
| Synthesis of results | 21 | Present the main results of the review. If meta-analyses done, include for each, confidence intervals and measures of consistency. | 9 |
| Risk of bias across studies | 22 | Present results of any assessment of risk of bias across studies (see Item 15). | 9 |
| Additional analysis | 23 | Give results of additional analyses, if done (e.g., sensitivity or subgroup analyses, meta-regression [see Item 16]). | 9 |
| **DISCUSSION** | | | |
| Summary of evidence | 24 | Summarize the main findings including the strength of evidence for each main outcome; consider their relevance to key groups (e.g., healthcare providers, users, and policy makers). | 10 |
| Limitations | 25 | Discuss limitations at study and outcome level (e.g., risk of bias), and at review-level (e.g., incomplete retrieval of identified research, reporting bias). | 13 |
| Conclusions | 26 | Provide a general interpretation of the results in the context of other evidence, and implications for future research. | 14 |
| **FUNDING** | | | |
| Funding | 27 | Describe sources of funding for the systematic review and other support (e.g., supply of data); role of funders for the systematic review. | None |

Appendix 2. Search Strategy of Medlinea

| Concept | Strategy | Result |
| --- | --- | --- |
| 1 | exp osteoporosis/ or bone rarefaction/ | 60,935 |
| 2 | (osteoporosis* or bone rarefaction*).ti.ab | 79,393 |
| 3 | 1 or 2 | 97,322 |
| 4^b^ | Abaloparatide*.ti,ab | 210 |
| 5^b^ | (BA058* or BIM-44058* or ITM-058*).ti.ab | 4 |
| 6 | 4 or 5 | 211 |
| 7 | 3 AND 6 | 195 |
| 8 | (clinical trial or randomized controlled trial).pt. | 948,312 |
| 9 | 7 AND 8 | 24 |

^a^Search date: Sep 2^rd^, 2022

^b^Abaloparatide and BA058 has no result in "MeSH Terms"

(osteoporosis[MeSH Terms] OR bone rarefaction[MeSH Terms] OR osteoporosis[Title/Abstract] OR bone rarefaction[Title/Abstract]) AND (Abaloparatide[Title/Abstract] OR BA058[Title/Abstract] OR BIM-44058[Title/Abstract] OR ITM-058[Title/Abstract]) AND (clinical trial[Publication Type] OR randomized controlled trial[Publication Type])

Appendix 3. Assessment tools in Meta-analysis

1. **Cochrane Risk Of Bias Assessment Tool (CROBAT)**

| Random sequence generation | Allocation concealment | Blinding of participants and personnel | Blinding of outcome assessment | Incomplete outcome data | Selective reporting | Other bias |
| --- | --- | --- | --- | --- | --- | --- |
|  |  |  |  |  |  |  |
|  |  |  |  |  |  |  |

Each question had 3 answers: “Low risk”, “Moderate” and “High risk”.

“Low risk” when detailed methods were founded in manuscript.

“Moderate” when declared blinded without detailed method.

“High risk” when found no relevant declaration.

1. Grading of Recommendations, Assessment, Development and Evaluation (GRADE)

| Risk of  Bias | Inconsistency | Indirectness | Imprecision | Publication bias | Plausible Confounding | Magnitude of effect | Dose-response gradient |
| --- | --- | --- | --- | --- | --- | --- | --- |
|  |  |  |  |  |  |  |  |
|  |  |  |  |  |  |  |  |

Each following question had 3 answers: “No serious risk”, “Serious risk” and “Very serious risk”.

Risk of Bias: “Serious risk” when sensitive analysis resulted in significant difference.

Inconsistency: “No serious risk” when I^2^≤50%; “Serious risk”when 50<I^2^≤75%; “Very serious risk” when I^2^>75%

Indirectness: Comprehensive consideration in combination with information.

Imprecision: “Serious risk” when P>0.05

Publication bias: “No serious risk” when Egger’s test P>0.05; “Serious risk”when 0.01<P≤0.05; “Very serious risk” when P≤0.01.

Plausible Confounding had 2 answers: “No” and “Yes”. It would be assessed by comprehensive consideration in combination with information.

Magnitude of effect had 3 answers: “No”, “Yes” and “Extremely” related to odds ratio (OR). “No” when 0.5<OR<2; “Yes” when 0.2<OR≤0.5 or 2≤OR<5; “Extremely” when OR≤0.2 or OR≥5.

Dose-response gradient had 2 answers: “No” and “Yes”. “Yes” when P value of dose-response related outcome ≤0.05.

There were 4 levels of quality: “High”, “Moderate”, “Low” and “Very low”. Evidence of RCTs were initially assessed as “High”. “Serious risk” would reduce 1 level of quality and “Very serious risk” would reduce 2 levels of quality. While “Yes” could promote 1 level of quality and “Extremely” could promote 2 level of quality.

Appendix4.1 Result of BMD baseline (Abaloparatide vs Placebo)*


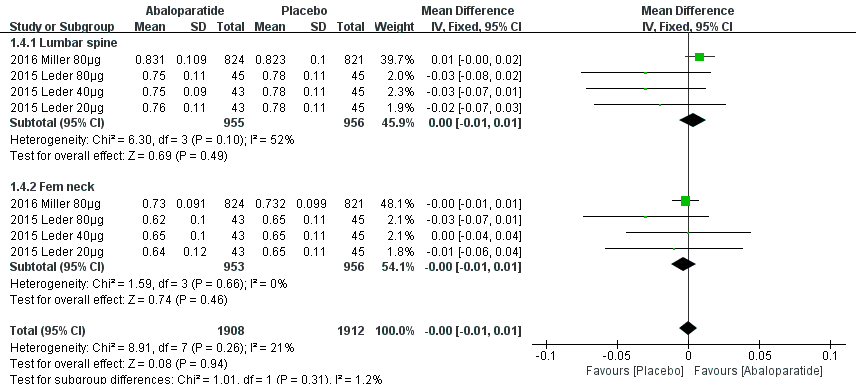


Appendix4.2 Result of BMD baseline (Abaloparatide vs Teriparatide)*


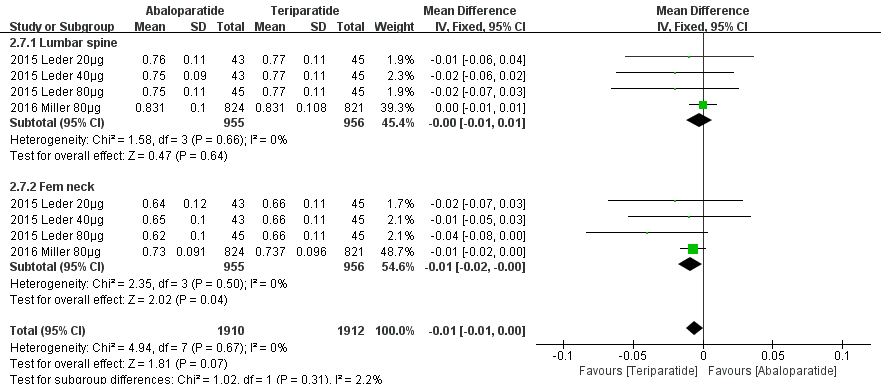


*Only one study by Miller et al. contained data of the total hip. We did not include a baseline BMD of the total hip for analysis in Appendix 4.1 and 4.2 due to insufficient data.

Appendix 5. Assessment result of CROBAT

| Author Year | Trail Number | Random sequence generation | Allocation concealment | Blinding of participants and personnel | Blinding of outcome assessment | Incomplete outcome data | Selective reporting | Other bias |
| --- | --- | --- | --- | --- | --- | --- | --- | --- |
| Leder 2015 | NCT00542425 | Simple Random Sampling. | Placebo, abaloparatide (20, 40, or 80 g), or teriparatide (20 g daily) randomized allocation. | Blinded | Blinded | No | No | No |
| Miller 2016 | NCT01343004 | Using a permuted-blocks design with a block size of 6 in a ratio of 1:1:1 to 1 of the 3 treatment groups. | Abaloparatide and placebo were administered with  identical pen injector devices under identical storage and pensing conditions. | Blinded | Blinded | No | No | No |
| Cosman 2017 | NCT01657162 | Simple random sampling in a ratio of 1:1:1. | Randomized to receive either blinded ABL-SC (80 µg) or blinded PBO | Blinded | Blinded | Yes | No | No |
| NCT01674621 | NCT01674621 | Simple Random Sampling. | The centralized BMD assessments and bone marker evaluations remained blinded to all treatment assignments | Blinded | Blinded | No | No | No |

## Appendix 6. Some Drugs for Postmenopausal Osteoporosis

| Drug | | Some formulations | Usual adult dosage^a^ | Cost^b^ |
| --- | --- | --- | --- | --- |
| **Bisphosphonates** | | | | |
| Alendronate – generic | | 5, 10, 35, 70 mg tablets;  70 mg/75 mL oral solution | Prevention: 5 mg PO once/d or 35 mg once/week | $21.10 |
|  | Fosamax (Merck) | 70 mg tablets | Treatment: 10 mg PO once/d or 70 mg once/week | 127.80 |
|  | Fosamax Plus D | 70 mg/2800 IU D_3_  70 mg/5600 IU D_3_ tablets | Treatment: 70 mg/2800 IU D_3_ or  Treatment: 70 mg/5600 IU D_3_ PO once/week | 173.30 |
|  | Binosto (Ascend) | 70 mg effervescent tablets | Treatment: 70 mg PO once/week^c^ | 300.00 |
| Ibandronate – generic | | 150 mg tablets; 3 mg/3 mL prefilled syringes and vials | Prevention: 150 mg PO once/month  Treatment: 150 mg PO once/month or 3 mg IV once every 3 month | 10.00^d^ |
|  | Boniva (Genentech) | 150 mg tablets; 3 mg/3 mL prefilled syringes | Prevention: 150 mg PO once/month  Treatment: 150 mg PO once/month or 3 mg IV once every 3 month | 527.40^e^ |
| Risedronate – generic  Sd Actonel (Allergan) | | 5, 35, 150 mg tablets^f^ | Prevention: 5 mg PO once/d, 35 mg once/week, or 150 mg once/month | 166.00 |
|  |  |  | Treatment: 5 mg PO once/d, 35 mg once/week, 75 mg 2 consecutive d/month, or 150 mg once/month | 369.30^i^ |
| Delayed-release – generic  sdAtelvia (Allergan) | | 35 mg delayed-release tablets | Treatment: 35 mg PO once/week | 171.60 |
|  |  |  | : Prevention:35 mg PO once/week | 266.60^i^ |
| Zoledronic acid^g^ – generic  sReclast (Novartis) | | 5 mg/100 mL IV solution | Prevention: 5 mg IV once every 2 year | 235.00^h^ |
|  |  |  | Treatment: 5 mg IV once/year | 1083.80^h^ |
| **Anti-RANK ligand antibody** | | | | |
| Denosumab – Prolia (Amgen)^i^ | | 60 mg/mL prefilled syringes | Treatment: 60 mg SC once every 6 months | 1278.80^j^ |
| **Parathyroid hormone analogs** | | | | |
| **Abaloparatide** – Tymlos (Radius)^k^ | | 3120 μg/1.56 mL prefilled pens | Treatment: 80 μg SC once/dl | 1966.40^j^ |
| Teriparatide – generic  Forteo (Lilly) | | 600 μg/2.4 mL prefilled pens | Treatment: 20 μg SC once/dl | 2475.00 |
|  |  |  | Prevention: 20 μg SC once/dl | 3597.80^i^ |
| **Selective estrogen receptor modulator** | | | | |
| Raloxifene – generic  Evista (Lilly) | | 60 mg tablets | Prevention: 60 mg PO once/d | 60.00 |
|  |  |  | Treatment: 60 mg PO once/d | 198.00^i^ |
| **Conjugated estrogens and selective estrogen receptor modulatorm** | | | | |
| Conjugated estrogens and bazedoxifene – Duavee (Pfizer) | | 0.45 mg/20 mg tablets | Prevention: 0.45 mg/20 mg PO once/d | 185.60 |
| **Sclerostin inhibitor** | | | | |
| Romosozumab-aqqg– Evenity (Amgen) | | 105 mg/1.17 mL prefilled syringes | Treatment: 210 mg SC once/month × 12 doses | 1825.00 |
| **Calcitoninn** | | | | |
| Calcitonin – generic | | 200 IU/spray | Treatment: 200 IU intranasally once/d | 74.90^o^ |

Referenced by: Pharmacotherapy for Postmenopausal Osteoporosis. JAMA. 2021 May 11;325(18):1888-1889. doi: 10.1001/jama.2020.13841.

^a^Dosage adjustments may be needed for renal or hepatic impairment.

^b^Approximate WAC for 30 days’ treatment at the lowest usual adult dosage or frequency. Cost of Duavee is based on dosage used for prevention.

WAC = wholesaler acquisition cost or manufacturer’s published price to wholesalers; WAC represents a published catalogue or list price and may not represent an actual transactional price.

Source: AnalySource® Monthly. June 5, 2020. Reprinted with permission by First Databank, Inc. All rights reserved. ©2020.[www.fdbhealth.com/policies/drug-pricing-policy](https://www.fdbhealth.com/policies/drug-pricing-policy).

^c^Should be dissolved in 4 oz of room-temperature plain water.

^d^Cost for tablets. Cost of one generic 3 mg/mL syringe is $240.00.

^e^Cost of one syringe.

^f^Risedronate is also available in a 30-mg tablet for treatment of Paget’s disease.

^g^Zoledronic acid is also available in a 4-mg formulation (Zometa, and generics) for treatment of hypercalcemia of malignancy, multiple myeloma, and bone metastases from solid tumors.

^h^Cost of one 5 mg/100 mL infusion bottle.

^i^Same dose with different duration between treatment and prevention which leads to different cost.

^j^Denosumab is also available in a 120 mg/1.7 mL formulation (Xgeva) for prevention of skeletal-related events in patients with bone metastases from solid tumors.

^k^Abaloparatide is a parathyroid hormone-related protein analog.

^l^Cumulative use for more than 2 years during a patient’s lifetime is not recommended.

^m^Conjugated estrogens are no longer recommended for first-line treatment of postmenopausal osteoporosis because of an increased risk of breast cancer, stroke, and venous thromboembolism.

^n^Because of safety concerns and limited evidence of efficacy, many experts no longer recommend use of salmon calcitonin.

^o^Cost of one 3.7-mL bottle.

Appendix 7. Indications for the use of Abaloparatide^*^

Indications for the use of Abaloparatide include:

1. In patients with BMD that meet one of the following conditions:
2. BMD T-score ≤ -3.5 based on BMD measurements from lumbar spine (at least two vertebral bodies), hip (femoral neck, total hip), or radius (one third radius site);
3. BMD T-score between -2.5 and -3.5, measured in the same manner, with a history of minor trauma from fracture;
4. BMD T-score between -1 and -2.5, measured in the same manner, along with a history of minor trauma due to fracture, or FRAX model predicted probability of fracture greater than or equal to 20% within 10 years, or a history of contraindication or intolerance to conventional osteoporosis treatment.
5. The patient's history of fracture includes at least one of these conditions:
6. Vertebral compression fracture;

(2) Fracture of the hip;

(3) Fracture of the distal radius;

(4) Fracture of the pelvis;

(5) Fracture of the proximal humerus;

3. Abaloparatide dosage: Formulation: 3120 µg/1.56 mL prefilled pens; usual adult dosage: 80 µg daily subcutaneous injection; maximum concentrations reached 0.5 h post-dose.

4. Abaloparatide duration: Treatment duration has not exceeded a total of 24 months of cumulative use of parathyroid hormone analogs (e.g., Teriparatide Injection, Forteo, Tymlos) during the patient’s lifetime.

^*^Referenced by FDA and UnitedHealthcare®

Reference:

1. Shirley M. Abaloparatide: First Global Approval. Drugs. 2017;77(12):1363-1368. doi: 10.1007/s40265-017-0780-7

2.Tymlos [package insert]. Waltham, MA: Radius Health, Inc.; April 2018.

3.Watts NB, Bilezikian JP, Camacho PM, Greenspan SL, Harris ST, Hodgson SF, et al. American Association of Clinical Endocrinologists Medical Guidelines for Clinical Practice for the diagnosis and treatment of postmenopausal osteoporosis. Endocr Pract. 2010;16(3):1-37. doi: 10.4158/ep.16.s3.1.

4.North American Menopause Society (NAMS). Management of osteoporosis in postmenopausal women: 2010 position statement of The North American Menopause Society. Menopause. 2010;17(1):25-54. doi: 10.1097/gme.0b013e3181c617e6.

5.Cosman F, de Beur SJ, LeBoff MS, Lewiecki EM, Tanner B, Randall S, et al. Clinician's Guide to Prevention and Treatment of Osteoporosis. Osteoporos Int. 2014;25(10):2359-81. doi: 10.1007/s00198-014-2794-2.

6.Hodsman AB, Bauer DC, Dempster DW, Dian L, Hanley DA, Harris ST, et al. Parathyroid hormone and teriparatide for the treatment of osteoporosis: a review of the evidence and suggested guidelines for its use. Endocr Rev. 2005;26(5):688-703. doi: 10.1210/er.2004-0006.

7.Hodsman A. Clinical practice guidelines for the use of parathyroid hormone in the treatment of osteoporosis. CMAJ. 2006;175(1):48. doi: 10.1503/cmaj.060624.

8.Florence R, Allen S, Benedict L, Compo R, Jensen A, Kalogeropoulou D, Kearns A, Larson S, Mallen E, O'Day K, Peltier A, Webb B. Diagnosis and treatment of osteoporosis. Bloomington (MN): Institute for Clinical Systems Improvement (ICSI); 2017 Jul. 62 p.

9.WHO FRAX tool: shef.ac.uk/FRAX. Accessed 9/12/2018.

10.Eastell R, Rosen CJ, Black DM, Cheung AM, Murad MH, Shoback D. Pharmacological Management of Osteoporosis in Postmenopausal Women: An Endocrine Society* Clinical Practice Guideline. J Clin Endocrinol Metab. 2019;104(5):1595-622. doi: 10.1210/jc.2019-00221.

Appendix 8. Funnel plots

Appendix 8.1 BMD Change(%) -24 weeks (Abaloparatide vs Placebo)*


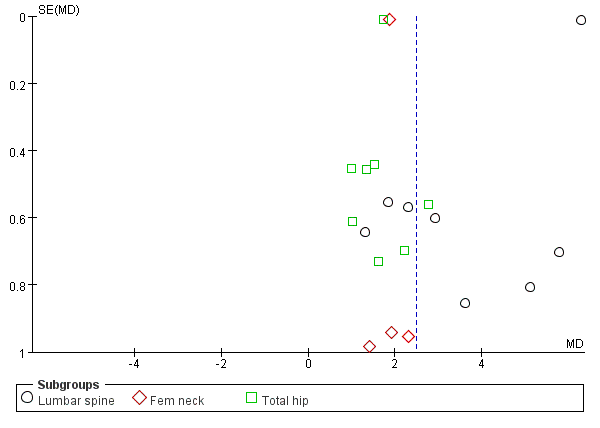


Appendix 8.2 BMD Change(%) -24 weeks (Abaloparatide vs Teriparatide)*


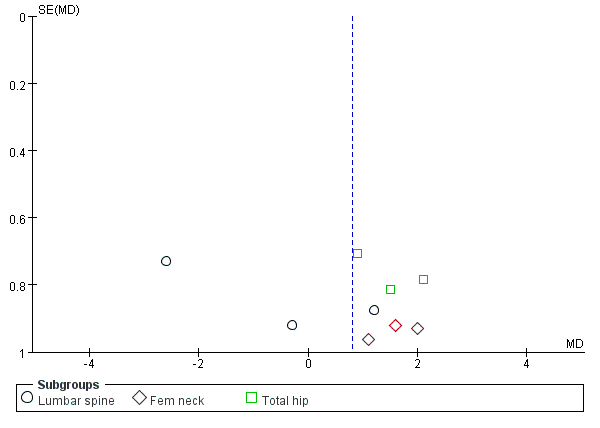


*Because of the I^2^ of BMD Change (%)-24 weeks up to 100% (Abaloparatide vs Placebo) and 73% (Abaloparatide vs Teriparatide), the funnel shape could not be presented in Appendix 8.1 and Appendix 8.2.

Appendix 8.3 Any adverse event (Abaloparatide vs Placebo)


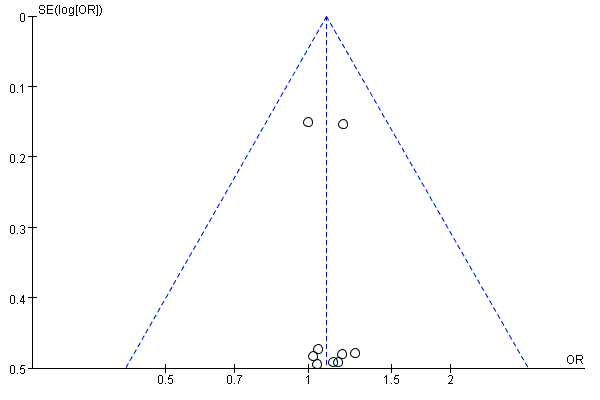


Appendix 8.4 Any adverse event (Abaloparatide vs Teriparatide)


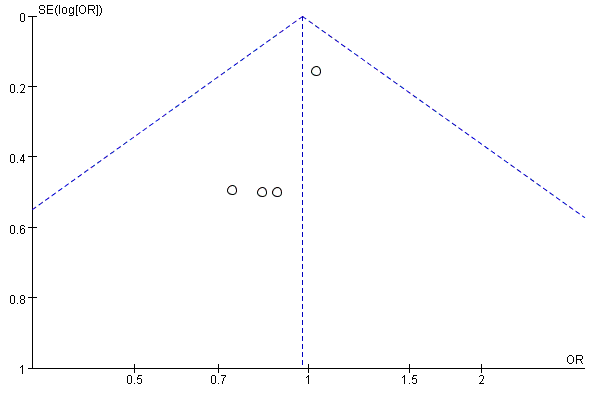


Appendix 8.5 Serious adverse events (Abaloparatide vs Placebo)


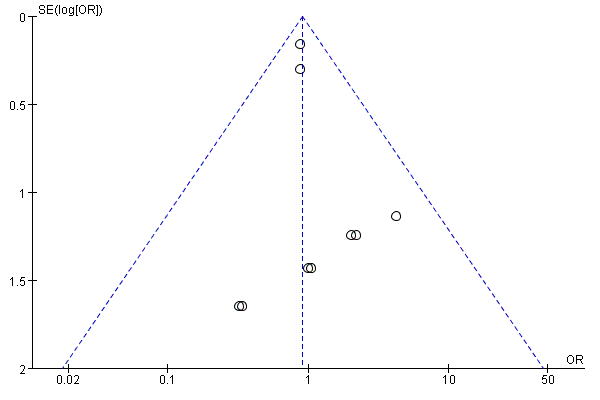


Appendix 8.6 Serious adverse events (Abaloparatide vs Teriparatide)


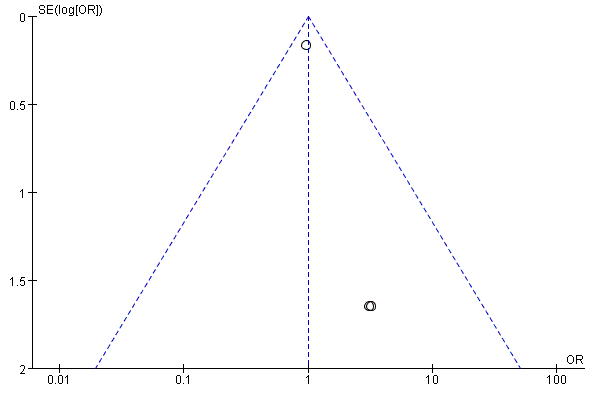

Supplement: Supplementary file 1 — Additional file 1: Appendix 1 PRISMA Checklist. Appendix 2 Search Strategy of Medline. Appendix 3 Assessment tools in Meta-analysis. Appendix 4 Result of baseline BMD. Appendix 5 Assessment result of CROBAT. Appendix 6 Some Drugs for Postmenopausal Osteoporosis. Appendix 7 Indications for the use of Abaloparatide. Appendix 8 Funnel plots [file 13018_2023_3595_MOESM1_ESM.doc]
